# Pre-Weaning Growth Hormone Treatment Reverses Hypertension and Endothelial Dysfunction in Adult Male Offspring of Mothers Undernourished during Pregnancy

**DOI:** 10.1371/journal.pone.0053505

**Published:** 2013-01-07

**Authors:** Clint Gray, Minglan Li, Clare M. Reynolds, Mark H. Vickers

**Affiliations:** Liggins Institute and Gravida, National Centre for Growth and Development, University of Auckland, Auckland, New Zealand; The University of Manchester, United Kingdom

## Abstract

Maternal undernutrition results in elevated blood pressure (BP) and endothelial dysfunction in adult offspring. However, few studies have investigated interventions during early life to ameliorate the programming of hypertension and vascular disorders. We have utilised a model of maternal undernutrition to examine the effects of pre-weaning growth hormone (GH) treatment on BP and vascular function in adulthood. Female Sprague-Dawley rats were fed either a standard control diet (CON) or 50% of CON intake throughout pregnancy (UN). From neonatal day 3 until weaning (day 21), CON and UN pups received either saline (CON-S, UN-S) or GH (2.5 ug/g/day)(CON-GH, UN-GH). All dams were fed ad libitum throughout lactation. Male offspring were fed a standard diet until the end of the study. Systolic blood pressure (SBP) was measured at day 150 by tail cuff plethysmography. At day 160, intact mesenteric vessels mounted on a pressure myograph. Responses to pressure, agonist-induced constriction and endothelium-dependent vasodilators were investigated to determine vascular function. SBP was increased in UN-S groups and normalised in UN-GH groups (CON-S 121±2 mmHg, CON-GH 115±3, UN-S 146±3, UN-GH 127±2). Pressure mediated dilation was reduced in UN-S offspring and normalised in UN-GH groups. Vessels from UN-S offspring demonstrated a reduced constrictor response to phenylephrine and reduced vasodilator response to acetylcholine (ACh). Furthermore, UN-S offspring vessels displayed a reduced vasodilator response in the presence of L-NG-Nitroarginine Methyl Ester (L-NAME), carbenoxolone (CBX), L-NAME and CBX, Tram-34 and Apamin. UN-GH vessels showed little difference in responses when compared to CON and significantly increased vasodilator responses when compared to UN-S offspring. Pre-weaning GH treatment reverses the negative effects of maternal UN on SBP and vasomotor function in adult offspring. These data suggest that developmental cardiovascular programming is potentially reversible by early life GH treatment and that GH can reverse the vascular adaptations resulting from maternal undernutrition.

## Introduction

Maternal undernutrition, a major risk factor for low birth weight has been shown to increase the risk of developing cardiovascular disease during adult-life in humans [1] and animal models [2], [3]. The concept of undernutrition during gestation or early life having adverse effects on the offspring’s health as an adult, suggests that disease or metabolic disorders can be ‘programmed’ in utero by a nutritional insult during critical periods of early development [1]. The fetal programming hypothesis suggests that a nutritional insult during development will adapt to the immediate environment causing permanent alterations in tissue architecture, cell number and function, rendering the offspring metabolically disadvantaged at times of dietary fluctuations as an adult [1], [4], [5].

Elevated resting blood pressure and increased risk of cardiovascular disease caused by prenatal undernutrition has been characterised by endothelial dysfunction [3], [6], tissue remodelling [7], reduced angiogenesis [8] and enhanced vascular superoxide production in adult offspring [9], [10]. Hypertensive offspring of rats fed a diet of reduced total calorific intake (30–50%) have impaired vasodilator responses to sodium nitroprusside in small mesenteric resistance vessels [6] and endothelium-dependant responses in aortic rings [11]. Similarly, offspring from maternally undernourished dams have also shown blunted response to ACh in mesenteric arteries indicative of a decreased endothelium-dependent vasodilation [12]. Furthermore, nitric oxide (NO) is one of the major bio-active vasodilator molecules and the constitutive production of nitric oxide within the vascular endothelium is important for determining basal arteriolar tone. Maternal undernutrition has also been reported to inhibit nitric oxide synthase activity [13]. However, the development of altered vascular function and endothelial dysfunction in adult offspring is still poorly understood. Therefore, dysfunction of the vascular endothelium could either contribute to the onset of hypertension, or develop as a consequence, thus it is unknown whether the reported changes such as endothelial dysfunction are a cause or result of hypertension.

The deleterious effects of maternal undernutrition on an offspring’s tissue development and subsequent programmed cardiovascular phenotype are largely thought to be permanent and irreversible. Previous studies have shown maternal undernutrition during pregnancy results in adult offspring hypertension [14], [15], endothelial dysfunction [12], obesity and altered metabolic profile as well as the reversal of these phenotypes by neonatal or maternal interventions [16]. Additionally, our previously published results have shown a complete reversal of programmed metabolic profile and later life obesity in offspring of undernourished mothers by neonatal leptin treatment [16]. Jackson and colleagues reported that maternal glycine supplementation reversed hypertension in offspring of maternal low-protein mothers [17]. Further intervention studies investigating the potential reversal of nutritionally-induced programmed hypertensive phenotypes have been shown in both nutritional [18] and pharmacological intervention studies [19], [20] in the low protein model. Nevertheless, comparatively few studies have investigated early life interventions in offspring of total calorie restricted mothers to ameliorate later life programming of hypertension.

Although the idea that growth hormone (GH) is critical for normal growth, maintenance of skeletal muscle mass and metabolic homeostasis is well accepted. Increasing attention has been directed towards the specific influences of GH on cardiac structure and function. There is now substantial evidence that GH exerts a direct, beneficial effect on the structure and function of the heart and vasculature [21], [22], [23]. Age-dependant decrease in GH has been associated with elevated blood pressure and subsequent GH treatment can reduce blood pressure in aged rats [24]. Furthermore, excess GH in patients suffering from acromegaly has been shown to increase ventricular wall thickness, leading to decreased systolic function and reduced peripheral vascular resistance [25], [26] and long term GH treatment to children born small for gestational age has shown a reduction in blood pressure [27]. Additionally, GH treatment in adults with GH deficiency (GHD) has been shown to reduce blood pressure and peripheral resistance and improvements in diastolic blood pressure have been reported following GH therapy of hypopituitarism in adults [28], [29]. In GHD patients, chronic substitution of GH results in increased rates of synthesis of NO and decreased peripheral arterial resistance. Studies have shown GH treatment may be beneficial by improving myocardial performance, and peripheral dilation by GH-induced increased circulating levels of insulin-like growth factor 1 (IGF-1) and up-regulating NO and cyclic guanosine monophosphate production [30], [31], [32]. Similar to clinical observations, we have previously shown that adult GH treatment in rats that were hypertensive as a consequence of maternal undernutrition normalises systolic blood pressure [33]. These studies provide evidence that GH may, in part, reduce the risk cardiovascular disease [34] and GH is therefore an important factor in the development, maintenance and function of the cardiovascular system.

To date, the potential of GH treatment as an intervention strategy during the period of early life developmental plasticity to prevent elevated BP and related vascular disorders in later life has not been well characterised. Utilising our well established model of maternal undernutrition to induce developmental programming, we aimed to investigate whether pre-weaning GH treatment plays a role in reversing the development of hypertension and perturbed vascular function during later life.

## Methods

All animal work was approved by the Animal Ethics Committee of the University of Auckland. We utilised a model of moderate undernutrition in the rat as described previously by us and others [35], [36], [37]. Female Sprague-Dawley rats (110 days of age, (n = 32)) were time-mated using an estrus cycle monitor (Fine Science Tools, USA). Upon confirmation of mating, two maternal dietary groups were established: (1) Controls (CON, N = 16): females maintained on a standard chow diet (Diet 2018, Harlan, USA) *ad-libitum* throughout pregnancy and lactation; (2) females fed at 50% of controls throughout pregnancy (UN, n = 16). All pregnant dams were weighed and had food intakes measured daily throughout pregnancy. Following birth, pups were weighed, had body lengths recorded and litter size was randomly adjusted to 8 pups to ensure standardized nutrition until weaning. Non-assigned pups were killed by decapitation. At birth, UN dams were fed a standard chow diet throughout lactation. Lactating dams had body weights and food intakes measured throughout the lactation period and pups were weighed every second day until weaning. At postnatal day 3, eight litters per maternal dietary group were randomly assigned to receive either saline or recombinant bovine growth hormone (rbGH, Cyanamid, USA) at a dose of 2.5 ug/g/day by subcutaneous injection until the time of weaning (day 22). This resulted in 4 treatment groups in a balanced 2×2 factorial design (CON-S, CON-GH, UN-S, UN-GH). Offspring weights were taken every second day until day 11 and every third day thereafter.

At weaning, male offspring were housed 2 per cage (2 per litter/treatment/maternal background) and fed the standard chow diet ad-libitum until the end of the trial (day 150). A minimum of 10 animals per offspring group were investigated. At postnatal day 140, systolic blood pressure was measured via tail cuff plethysmography as described previously [33] (Model 179, IITC Life Science Inc, USA) and heart/respiration rates assessed via pulse oximetry as detailed below. At postnatal day 150, animals were fasted overnight and killed by decapitation following anaesthesia with sodium pentobarbitone (60 mg/kg, IP).

### Systolic Blood Pressure (SBP), Oxygen Saturation, Heart and Respiration Rates

Systolic blood pressure (SBP) at day 150 was recorded by tail cuff plethysmography according to the manufacturer’s instructions (Model 179 with an automatic cuff inflation pump (NW20), IITC, Life Science, Woodland Hills, CA) as previously described [38], [39]. The IITC system has been validated against both telemetry and direct blood pressure measurements via cannulation in the rodent and allows recording at lower ambient temperatures compared to other systems [40]. Rats were restrained in a clear plastic tube in a pre-warmed room (25–28 C). After the rats had acclimatised (10–15 min) the cuff was placed on the tail and inflated to 240 mmHg. Pulses were recorded during deflation at a rate of 3 mmHg/s and reappearance of a pulse was used to determine SBP. A minimum of three clear SBP recordings were taken per animal and the coefficient of variation for repeated measurements was <5%. Blood oxygen (O_2)_ saturation, heart rate and breath rate were monitored using a small animal pulse oximeter collar placed around the neck (MouseQx +Plus; Starr Life Sciences, USA.). After an initial habituation to wearing the collars, baseline measurements were recorded every 3 mins for 60 sec. over a 15 min time period. Collar displacement artefacts were excluded from the analysis. Experiments were performed on freely mobile rats fed *ad libitum*.

### Vascular Studies

The mesenteric bed was removed and placed in a dissecting dish containing physiological salt solution (PSS) (119 mM NaCl, 4.7 KCl, 2.5 CaCl_2_, 24 NaHCO_3_, 1.18 KH_2_PO_4_, 1.2 MgSO_4_, 0.01 EDTA, 5.5 glucose) on ice. Third-order mesenteric vessels (<300 µm) were isolated from the mesenteric vascular bed and connecting tissue under a dissecting microscope. Vessel segments were then mounted on a pressure myograph system (Living System, Burlington, VT, USA.). Briefly, the vessel was placed on two glass microcannulae, secured with nylon suture and vessel length was adjusted without stretch and parallel. Intraluminal pressure was then raised to 100 mmHg and the artery was unbuckled by adjusting the cannulae. Functional integrity was assessed with five 1 min washes with PSS and pre-constriction with phenylephrine (PE) (concentration equal to 80% of maximal response; pEC80). Vessels failing to produce constriction were considered non-viable and not utilised in the study. Where vessels were pre-constricted to pEC80 during repeated pharmacological administration, vessels failing to consistently reproduce consistent constriction (pEC80) were also considered non-viable and substituted with freshly excised tissue.

Pressure-diameter curves were obtained by increasing intraluminal pressure in 10 mmHg steps between 10 and 90 mmHg and external diameters were measured at each pressure. From these results, pressure diameter relationships were calculated as percentage change in initial diameter at 10 mmHg. Following an equilibration period in PSS, further vascular studies were studied in vessel segments pressurized to 70 mmHg following the equilibration period of 30 min or cessation of basal vascular activity, which ever was sooner at 37°C in PSS gassed with a mixture of 95% O_2_ and 5% CO_2_. Cumulative concentration response curves were constructed for the α1-adrenoceptor agonist PE (1 nM to 100 uM). Changes in diameter at each PE concentration were compared to initial vessel diameter as % constriction, and then normalised as % maximum constriction. Following pre-constriction with PE –log concentration equal to 80% of maximal response, cumulative concentration curves were constructed with the endothelium-dependant vasodilator acetylcholine (ACh; 0.1 nM to 1 mM). Changes in diameter at each ACh concentration were compared to initial vessel diameter after pre-constriction with PE, and then normalised as percentage relaxation.

The current study investigated the 3 main mediators responsible for endothelium-dependent relaxation (NO, PGI_2_ and EDHF). To block NO production and soluble guanylyl cyclase activity, the non-specific NO synthase inhibitor L-NG-Nitroarginine Methyl Ester (L-NAME, 100 µM) and the highly selective inhibitor of soluble guanylyl cyclase 1H- [1], [2], [4]oxadiazolo[4,3-a]quinoxalin-1-one (ODQ, 5 µM) were used. Concentrations of L-NAME and ODQ were chosen from previously reported results showing consistent inhibitory effects of L-NAME [41] and ODQ [42], [43] on vasodilatory responses in isolated vessels. Indomethacin (INDO, 10 µM) was used to investigate the contribution of vasodilators derived from the cyclooxygenase pathway (e.g. prostacyclin). Concentrations of INDO (10 µM) were chosen from previously reported results showing the effects of INDO on vasoreactivity in isolated vessels [44]. The role of gap junctions and EDHF activity were investigated using the putative gap junction inhibitor carbenoxolone (CBX, 100 µM) [45] and ATP-type Ca+2-activated K+ channel blocker apamin (30 µM) [46] and intermediate-conductance Ca+2-activated K+ channel blocker TRAM-34 (1 µM) [47]. Both apamin and TRAM-34 in the presence of L-NAME (100 µM), ODQ (5 µM) and INDO (10 µM) were analysed to ensure that relaxation to Ach in the presence of L-NAME and INDO was a true EDHF response. Inhibitors were used individually and in combination where stated.

### Statistical Analysis

Statistical analysis was performed by two-way repeated measures factorial ANOVA with Bonferroni post-hoc test where appropriate; linear regression analysis was used to analyse diameter/pressure relationships. Concentration-relaxation curves were constructed using Prism software (GraphPad Software Inc., La Jolla, CA, USA.) Data are shown as means ± SEM. A probability of P<0.05 was accepted as statistically significant.

## Results

### Birth Weights and Body Growth

**Figure 1:**
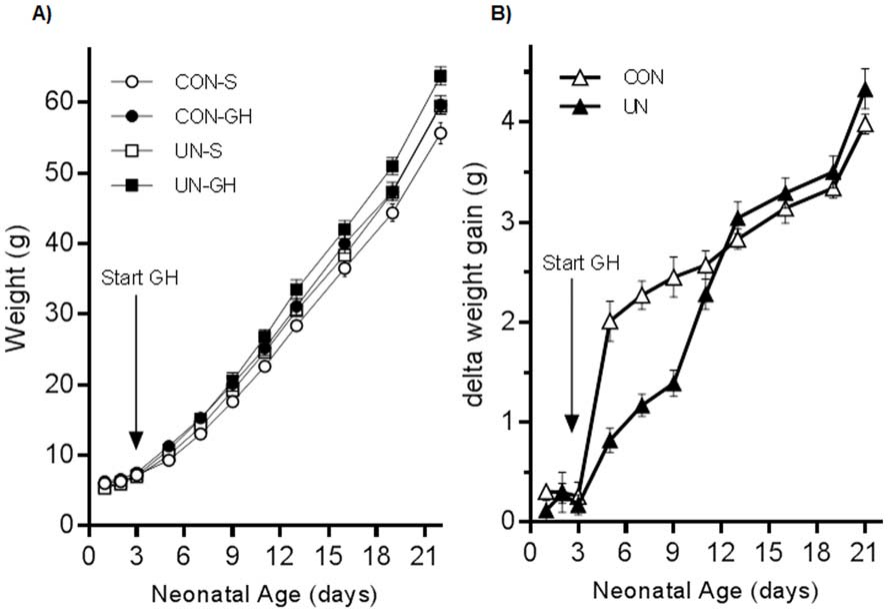
Absolute neonatal weights (a) and delta weight change in GH-treated neonates compared to saline treated offspring (b) from day 3 until day 21. Data are means ± SEM, n = minimum of 8 litters per treatment group. P<0.0001 for effect of GH versus saline.

Moderate maternal undernutrition resulted in a significant reduction in birth weight (CON 6.1±0.05 g, UN 5.4±0.05, p<0.0001) and birth length (nose-anus: CON 47±0.2 mm, UN 44±0.2, p<0.001 for maternal diet effect). There were no differences in litter size between CON and UN groups (data not shown). There was a clear body weight response to GH treatment in both CON and UN groups (Figures 1a–b). Of note there was a delayed response to GH treatment in UN-GH offspring but this normalised to that of CON-GH group by the end of treatment. At weaning (day 22), UN offspring had shown significant catch-up growth compared to CON offspring. GH significantly increased body weight in both groups (CON-S 55.7±0.8, CON-GH 59.7±0.8, UN-S 59.5±0.9, UN-GH 63.9±1.0, p<0.05 for effect of maternal diet and GH treatment). At the end of the study period, absolute body weights were not significantly different across the groups. There were no effects of maternal diet or pre-weaning GH treatment on adult body lengths, kidney or adrenal weights (data not shown). Absolute heart weight was increased in UN-S offspring compared to CON offspring and was normalised in UN-GH offspring (CON-S 1.86±0.06 g, CON-GH 1.88±0.06, UN-S 2.10±0.08, UN-GH 1.86±0.05, p<0.05 for UN-S versus all other groups, maternal diet x GH treatment interaction p<0.05). When normalised to body weight, there were no differences in heart weight across any of the treatment groups.

### Systolic Blood Pressure (SBP) Recordings

**Figure 2:**
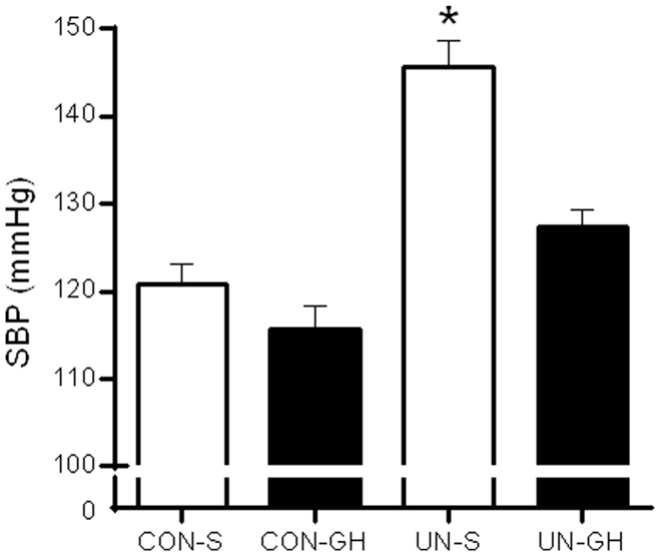
Systolic blood pressure (SBP) at postnatal day 140 in male offspring as quantified via tail-cuff plethysmography. *p<0.001 for UN-S versus all other groups. Maternal diet and GH treatment effect p<0.001. Maternal diet x GH treatment interaction p<0.005. Data are means ± SEM, n = 10 per group.

SBP at day 140 was significantly increased in UN-S male offspring when compared to CON-S and CON-GH offspring and normalised in UN-GH animals (Figure 2). Pre-weaning growth hormone treatment did not have an effect on SBP in CON-GH offspring when compared to CON-S animals. Prenatal diet or GH treatment did not have any effect on percentage oxygen (O_2_) saturation (UN-S 95.83±0.31, CON-S 95.63±0.35, CON-GH 95.8±0.27, UN-GH 95.9±0.24, heart rate (UN-S 315±6, CON-S 324±10, CON-GH 319±9, UN-GH 311±17 or breathing rate (UN-S 100±1.83, CON-S 98±1.9, CON-GH 98.9±2.1, UN-GH 101±1.5).

### Mesenteric Vessel Responsiveness to PE

**Figure 3:**
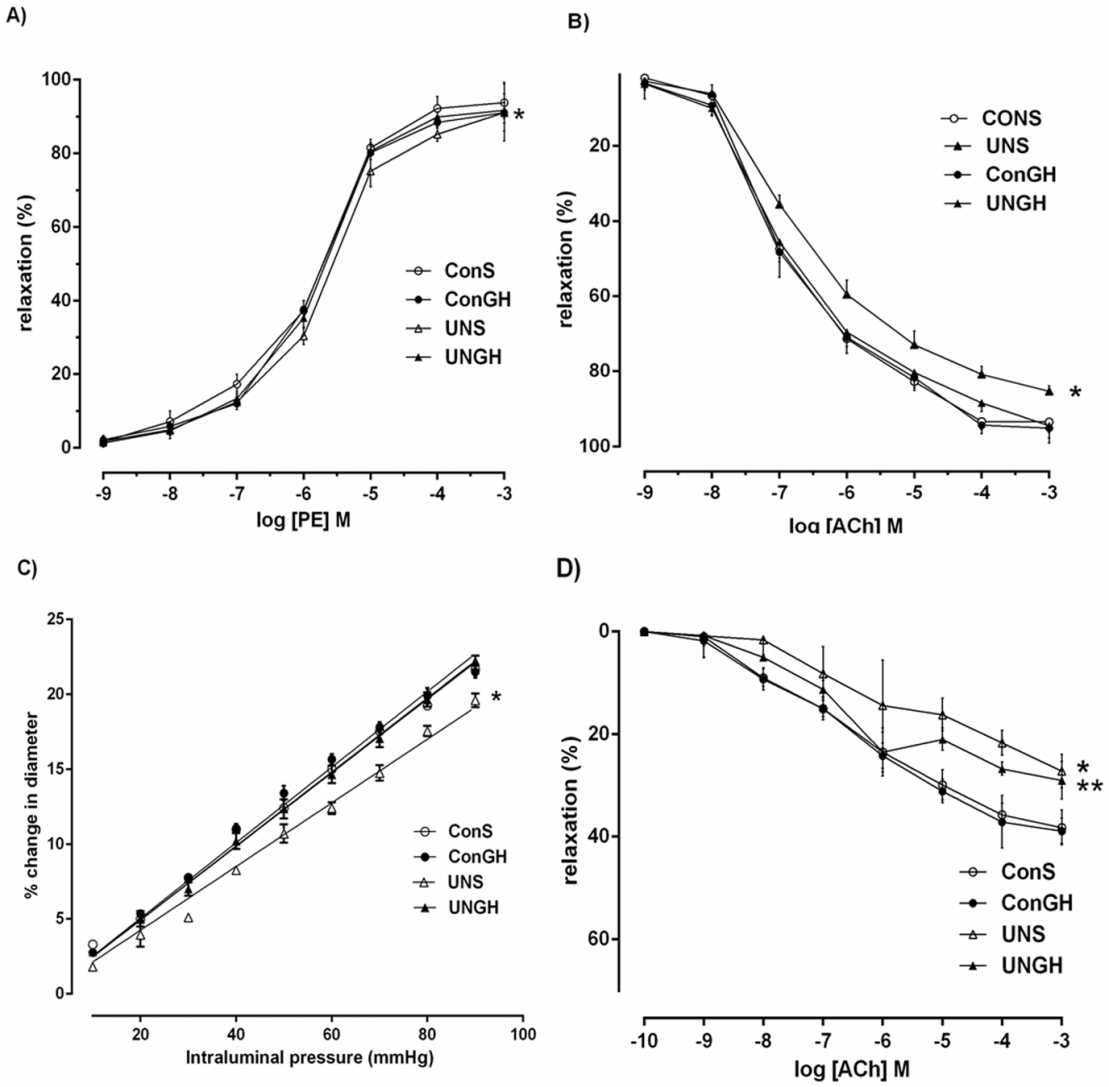
(a) Mesenteric vessel responsiveness following phenylephrine (PE) treatment, measured as % change from initial resting diameter and normalised as % maximum constriction in CON-S (○), CON-GH (•), UN-S (□) and UN-GH (▪) adult male offspring. * p<0.001 for overall effect of UN-S versus all other groups. (b) Mesenteric vessel responsiveness following cumulative additions of vasodilator acetycholine (ACh) and expressed as % change from initial resting diameter after pre-constriction with PE (10 µM). * p<0.001 for overall effect of UN-S versus all other groups. (c) Mesenteric vessel myogenic responsiveness to pressure, measured as % change from initial vessel diameter at 10 mmHg. * p<0.001 for UN-S versus all other groups using linear regression analysis. (d) Mesenteric vessel responsiveness following cumulative addition of vasodilator ACh measured as % change from initial resting diameter after pre-constriction with PE (10 µM) in the presence of L-NAME (100 µM) and ODQ (5 µM). * p<0.001 for UN-S versus CON-S and CON-GH; ** p<0.001 for overall difference between UN-GH and CON-S and CON-GH. All data are means ± SEM, n = 10 per group. L-NAME: Nψ-nitro-L-arginine methyl ester; ODQ: 1H- [1], [2], [4]oxadiazolo[4,3-a]quinoxalin-1-one.

PE produced a concentration-dependant vasoconstriction in all vessels. Mesenteric vessel responsiveness to PE was significantly reduced (Figure 3a) in UN-S offspring when compared with CON-S and CON-GH and normalised in UN-GH offspring vessels (pEC50; UN-S 5.55±0.03, CON-S 5.79±0.02, CON-GH 5.72±0.02, UN-GH 5.71±0.02, P<0.001). Pre-weaning GH treatment did not have an effect on constriction in CON-GH when compared to CON-S vessels.

### Mesenteric Vessel Responsiveness to ACh

ACh produced a concentration-dependant vasodilatation in all vessels. Pre-weaning growth hormone treatment was observed to normalise mesenteric vessel responsiveness to ACh (Figure 3b). UN-S mesenteric vessel responsiveness to ACh was significantly reduced when compared to CON-S, CON-GH and UN-GH vasomotor activity (% maximum response; UN-S 85.3±0.41, CON-S, 93.4±0.9, CON-GH 95.1±0.8, UN-GH 94.5±1.4, P<0.001). No differences were observed between CON-S and CON-GH vessels.

### Vessel Diameter-pressure Relationship

A pressure-dependant vasodilatation was observed in all vessels. Second order mesenteric vessels (Figure 3c) from UN-S male offspring displayed a decreased myogenic reactivity to increased pressure when compared to CON-S and CON-GH offspring and normalised in UN-GH male offspring (Slope; UN-S 0.2126, CON-S 0.2471, CON-GH 0.2523, UN-GH 0.2460 P<0.001). Pre-weaning GH treatment did not have an effect on the diameter-pressure relationship in CON-GH offspring vessels when compared to CON-S group.

### ACh-induced Relaxation in Vessels Incubated with L-NAME/ODQ

In the presence of L-NAME (100 µM) and ODQ (5 µM), ACh-induced relaxation was observed in a concentration-dependant manner in all vessels (Figure 3d). Ach-induced relaxation was significantly reduced in UN-S mesenteric vessels when compared to CON-S and CON-GH vessel responsiveness (% maximum response; UN-S, 27.2±1.0 *Vs*. CON-S 38.2±1.07, CON-GH 38.9±0.78, UN-GH 29±1.82, P<0.001). A clear intermediary effect of pre-weaning GH treatment can be observed in the UN-GH group. Pre-weaning GH treatment did not have an effect on CON-S *vs.* CON-GH vessel responsiveness.

### ACh-induced Relaxation in Vessels Incubated with INDO

**Figure 4:**
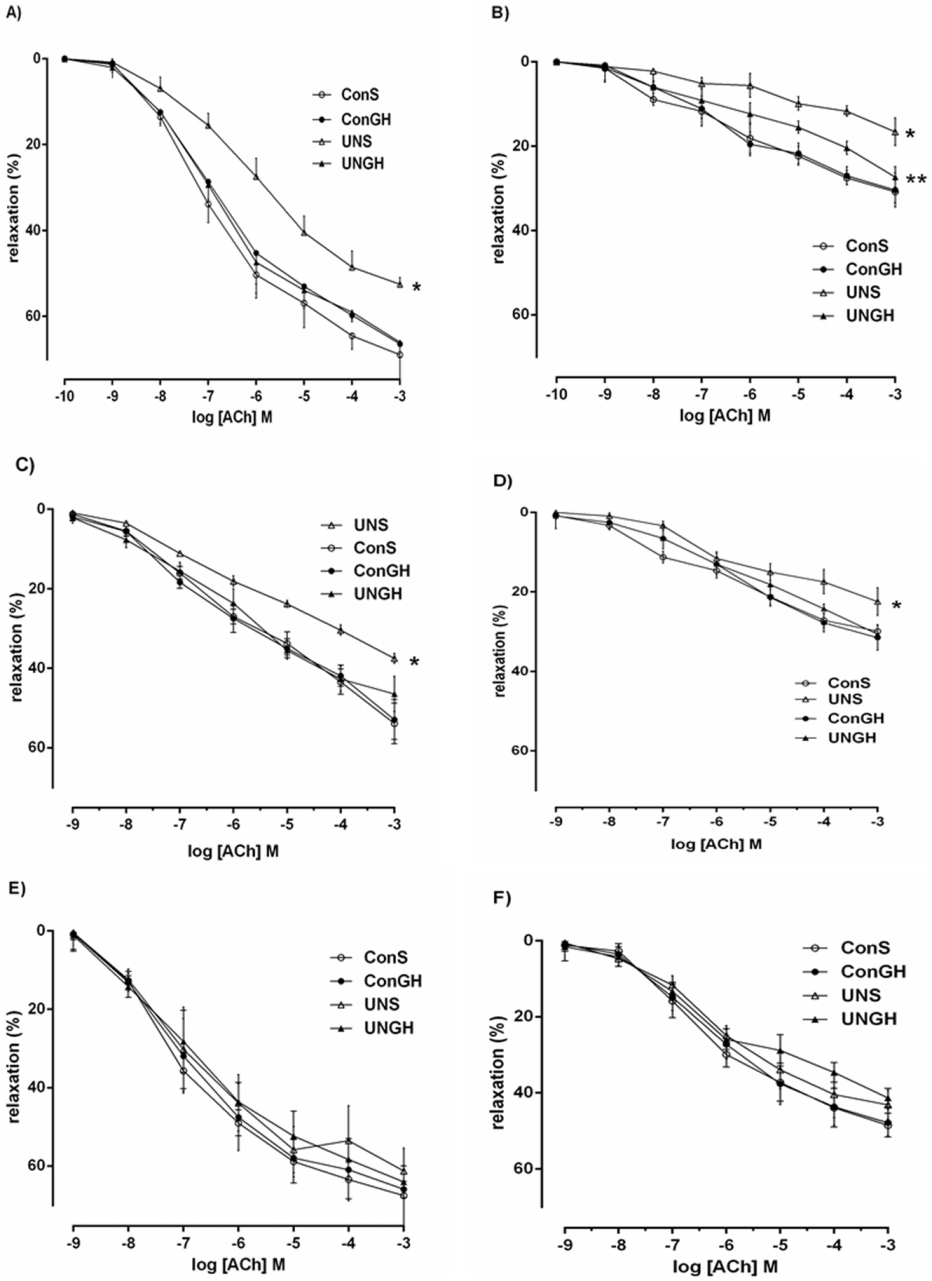
(a) Mesenteric vessel responsiveness following cumulative additions of vasodilator acetylcholine (ACh) measured as % change from initial resting diameter after pre-constriction with PE (10 µM) in the presence of CBX (100 µM). * p<0.001 for UN-S versus all other groups. (b) Mesenteric vessel responsiveness following cumulative additions of vasodilator ACh measured as % change from initial resting diameter after pre-constriction with PE (10 µM) in the presence of L-NAME (100 µM), INDO (10 µM) and CBX (100 µM). * p<0.001 for UN-S versus all other groups. ** p<0.001 for UN-GH versus UN-S. (c) Mesenteric vessel responsiveness following cumulative additions of vasodilator ACh measured as % change from initial resting diameter after pre-constriction with PE (10 µM) in the presence of TRAM-34 (1 µM) and Apamim (30 µM). * p<0.001 for UN-S versus all other groups. (d) Mesenteric vessel responsiveness following cumulative additions of vasodilator ACh measured as % change from initial resting diameter after pre-constriction with PE (10 µM) in the presence of TRAM-34 (1 µM), Apamim (30 µM), L-NAME (100 µM) and INDO (10 µM). * p<0.001 for UN-S versus all other groups. (e) Mesenteric vessel responsiveness following cumulative additions of vasodilator ACh measured as % change from initial resting diameter after pre-constriction with PE (10 µM) in the presence of INDO (10 µM). (f) Mesenteric vessel responsiveness following cumulative additions of vasodilator ACh measured as % change from initial resting diameter after pre-constriction with PE (10 µM) in the presence of L-NAME (100 µM) and INDO (10 µM). All data are means ± SEM, n = 10 per group. L-NAME: Nψ-nitro-L-arginine methyl ester; INDO: indomethacin; CBX: carbenoxolone disodium; TRAM-34: Ca^2+^-activated K^+^ channel blocker.

In the presence of indomethacin (10 µM) ACh-induced vasodilatation was observed to be reduced in all groups. However, ACh concentration-dependant relaxation was not different between groups (Figure 4e).

### ACh-induced Relaxation in Vessels Incubated with INDO and L-NAME

Additionally mesenteric vessel responsiveness in the presence of L-NAME (100 µM) and INDO (10 µM) were also observed to be not different between groups (Figure 4f).

### ACh-induced Relaxation in Vessels Incubated with CBX

In the presence of CBX (100 µM), ACh-induced vasodilatation was observed to be reduced in all groups. UN-S vessel responsiveness was significantly reduced when compared to CON-S, UN-GH, and CON-GH groups (% maximum response; UN-S 52.5±0.47, UN-GH 66±1.10, CON-S 68.9±1.23, CON-GH 66.4±1.42), P<0.001). Pre-weaning GH treatment completely normalised the significantly reduced Ach-induced vasodilatation observed in UN-S offspring mesenteric vessels (Figure 4a). CON-S and CON-GH were not different from each other.

### ACh-induced Relaxation in Vessels Incubated with CBX & L-NAME

In the presence of CBX (100 µM), and L-NAME (100 µM), ACh-induced vasodilatation was observed in all groups (Figure 4b). Vessel responsiveness was not different between UN-GH, CON-S and CON-GH up to -6Log [ACh] M (1 uM). UN-GH vessel responsiveness was significantly (P<0.001) improved when compared to UN-S offspring vessels, with a clear intermeadiatary effect of pre-weaning GH can be observed in the UN-GH group (% maximum response; UN-GH 27.3±0.77, UN-S 16.6±1.03, P<0.001). CON-S and CON-GH were not different from each other.

### ACh-induced Relaxation in Vessels Incubated with TRAM-34 & Apamin

In the presence of TRAM-34 (1 µM) and Apamin (30 µM) mesenteric vessels produced a reduced concentration-dependant relaxation to Ach in all groups (Figure 4c). UN-S vessel responsiveness was significantly reduced when compared to CON-S and CON-GH offspring and normalised in UN-GH male offspring(% maximum response; UN-S 37.5±1.24, CON-S 53.8±1.80, CON-GH 52.8±1.66, UN-GH 46.4±1.482, P<0.001). Pre-weaning GH treatment did not have an effect on the vessel reactivity in CON-GH offspring when compared to CON-S offspring.

### ACh-induced Relaxation in Vessels Incubated with TRAM-34, Apamin, L-NAME & INDO

The combination of TRAM-34 (1 µM) and Apamin (30 µM) in the presence of L-NAME (100 µM) and INDO (10 µM) induced a significant reduction in Ach-induced vasodilatation in all groups (Figure 4d). Pre-weaning growth hormone treatment improved vessel responsiveness in UN-GH offspring vessels when compared to UN-S vessels (% maximum response; UN-GH, 30.6±0.46, UN-S 22.4±1.15, P<0.001). Vasodilatory response did not differ between CON-S and CON-GH groups (% maximum response; CONT-S 29.8±0.51, CONT-GH 31.5±1.13) in mesenteric vessels.

## Discussion

In the present study, we investigated the effects of pre-weaning GH treatment on blood pressure, vascular function and associated alterations in cardiovascular control during adult life. Consistent with published data we also report low birth weight, catch-up growth, and hypertension in UN-S male offspring and completely normalised blood pressure by growth hormone treatment (day 3–21) in UN-GH offspring. Using the technique of small vessel pressure myography, we have demonstrated that daily treatment of growth hormone reduces blood pressure and improves vascular responsiveness in adult offspring from dams that were undernourished during pregnancy. Furthermore, our results indicate that impaired vascular function and hypertension in UN-S animals and the beneficial effects of early life GH treatment are likely to be due to alterations in one or both mechanisms of endothelium-derived hyperpolarizing factor (EDHF) and NO mediated vasodilatation.

Our first finding that GH appears to play a beneficial role in cardiovascular development is supported by a reduction of blood pressure in GH treated offspring with pre-weaning GH treatment reducing adult blood pressure on average ∼18 mmHg compared to hypertensive UN-S offspring. Previous studies have also shown the beneficial effects of GH treatment reducing age-related hypertension due to reduced systemic GH levels in rats [24]. Moreover, Yang *et al.* showed that GH treatment may improve cardiac function by both increased myocardial contractility and decreased peripheral vascular resistance in the rat heart [48]. Our initial evidence, when combined with evidence from other studies, supports the hypothesis that pre-weaning GH treatment may reverse the programming of adult cardiovascular function. We would therefore suggest that GH is an important mediator of early postnatal growth and development of the cardiovascular system.

Our second finding that gap junction uncoupler, CBX, markedly inhibited endothelium-dependent mediated relaxation in mesenteric vessels of UN-S offspring which was completely reversed in GH treated offspring provides further evidence that GH can be beneficial to the cardiovascular development of neonate. Inhibition of vasodilatation by CBX in the UN-S offspring vessels to <40% of maximal response is similar to the effects of CBX on vasodilatation previously reported by Goto *et al.* in rat mesenteric arteries [49]. Although the relative contribution of EDHF to ACh-induced relaxation may be greater in smaller vessels, studies have demonstrated that the EDHF-mediated hyperpolarization is impaired and evidence of altered vascular gap junction and EDHF function has been observed in rat models of hypertension, diabetes, and maternal undernutrition. In the presence of L-NAME, indomethacin, apamin and TRAM-34 relaxations were significantly reduced in UN-S and completely reversed by GH treatment. However, the relaxation to ACh was not completely abolished at concentrations above 10 µM. This raised the possibility of the remaining relaxation being an unknown factor, independent of NO, prostanoids and hyperpolarisation of the endothelial cells. This could be due to incomplete blockade of the calcium-activated potassium channels, but is unlikely considering the concentrations of drugs used in the present study are consistent with those used previously [50], [51]. The present data show that EDHF-mediated hyperpolarization and therefore gap junction transfer of EDHF components may play an important role in the development origins of hypertension in the current model. Additionally, it raises the important possibility that the impaired EDHF-mediated responses in UN-S offspring may be associated with reduced gap junction, myoendothelial gap junction distribution and/or function which may be improved by GH hormone treatment during development.

GH hormone has been reported to be primarily acting through a NO-dependant pathway associated with increased plasma IGF-I concentrations. GH treatment and subsequent increase in IGF-1 levels have been shown to improve endothelial dysfunction in rats [24], [26], [31]. Although plasma IGF-I levels were not measured in the current study, it has been previously shown that circulating IGF-1 levels are reduced at birth in the offspring of undernourished mothers [52]. The current study also suggests that GH treatment during postnatal development is affecting NO-dependant pathways. If NO production was blocked by L-NAME and ODQ, relaxation to ACh was significantly reduced in the UN-S, and an improved response observed in UN-GH offspring vessels, suggesting an impaired EDHF and to a lesser extent prostaglandin vasodilatation response in UN-S animals. Furthermore, whilst not capable of a full relaxation, following incubation with indomethacin or L-NAME and indomethacin, indicates that EDHF may be the prevalent relaxing factor in this strain, sex and vessel bed.

When NO and prostaglandin pathways were blocked, EDHF and other factors were capable of fully compensating the resulting reduction in vasoresponsiveness associated with the NO and prostaglandin pathways. However, these data do not necessarily suggest that the relaxation to ACh is mediated by EDHF rather than NO in the UN animals. This is consistent with current understanding, that EDHF is the largest contributor to vasodilatation in resistance vessels and we provide evidence that indicate EDHF is an essential functional pathway involved in the improved vascular responses in UN-GH and the perturbed vasoresponsiveness observed in UN-S offspring.

Sex-specific effects are often observed in studies related to developmental programming. The current study utilised males only due to logistical constraints and the possible confounder of stage of estrus on primary outcome measures. Although estrus stage has been shown to impact on daily variations in blood pressure [53], the data from the current study clearly indicate that further work in staged females is warranted to examine sex specific effects using this experimental paradigm. Additionally, consideration of the non-specificity of CBX must be given. CBX has been shown to have non-specific effects on varied ion channels and cellular processes, although conflicting reports exist [54], [55], [56]. We, like others opted to use CBX in the current study as results showing the inhibitory effects of CBX on gap junctions in isolated tissues have been widely reported [57], [58], [59].

Our findings further support the hypothesis that the more specific action of GH on cardiovascular development may primarily be mediated by changes in EDHF transfer between the endothelial cell and vascular smooth muscle via increased gap junctions and/or calcium activated potassium channel distribution and function. Since, reduced gap junction transfer, being related to the functional EDHF response of the vessel in the UN-S may contribute towards this reduction in EDHF activity, providing less opportunity for endothelial and smooth muscle cell communication [60], [61]. Possible mechanisms thought to be involved in role of GH on cardiovascular responses could be due to an increased bioavailability of NO and increased EDHF component combined due to the eutrophic remodelling and alterations in vessel wall components such as; elastin and vascular smooth muscle [62]. Vascular remodelling has been shown to involve; a hypertrophy or hypotrophy, of vascular wall components, ultimately reorganising vasoresponsive pathways [63], [64]. We feel that the current study provides evidence of a reorganisation of vasodilator pathways and possible structural remodelling are at least partly responsible for an increase in blood pressure, perturbed vascular function in UN-S offspring and the prevention of this phenotype by pre-weaning GH treatment.

In conclusion, the timing of pre-weaning GH treatment in the current study, from day 3–21, was carried out whilst the greatest period of postnatal developmental plasticity and our results suggest that pre-weaning GH treatment is capable of reversing the programmed effects observed in UN-S male offspring. Elevated GH *in vivo* may act as a developmental mediator, facilitating an increase in endothelial & vascular smooth muscle cells, compensating for the seemingly adverse vascular development in UN male offspring. However, further work needs to be completed to elucidate the specific mechanisms involved. It would be of great interest to investigate the proposed structural remodelling within the vasculature of UN-S and UN-GH offspring. Nevertheless, our findings show that maternal undernutrition impairs EDHF and to a lesser extent NO-dependant dependent hyperpolarization. Ultimately leading to a loss of functional integrity of the vascular wall and having a detrimental effect on the maintenance of peripheral blood flow and subsequent arterial blood pressure in UN adult offspring which can be reversed by pre-weaning GH treatment.
